# Complete heart block in a young adult with non-isolated congenitally corrected transposition of the great arteries: Case report

**DOI:** 10.1016/j.amsu.2022.103500

**Published:** 2022-03-14

**Authors:** Raid Faraj, Abakar Bachar, Oussama Sidaty, Asmaa Bouamoud, Zineb Fassi Fehri, Fatima-zahrae Chrifi, Fatima Chikhi, Ibtissam Fellat, Rachida Amri, Mohamed Cherti

**Affiliations:** Mohammed V University, Rabat, Morocco

**Keywords:** Corrected transposition of the great arteries, Complete heart block, Ventricular septal defects, Conduction disorders

## Abstract

**Introduction and importance:**

Congenitally corrected transposition of the great arteries (ccTGA) or L-looped transposition of the great arteries (L-TGA) is a very rare and complex form of congenital heart disease. The majority of patients with ccTGA have at least one or more associated congenital heart disorders, essentially ventricular septal defects. Patients with ccTGA can remain asymptomatic for a long time and the diagnosis can sometimes be made late in life at the stage of complications.

**Case presentation:**

Here, we report a rare case of a 19-year-old patient, with no medical or surgical history, presenting a complete heart block as initial presentation of a ‘’non-isolated’’ ccTGA. The diagnosis is made essentially by echocardiography.

This case aims to show diagnostic difficulties of this rare congenital heart disease and be aware of the risk of its relative complications.

## Highlights

1) Congenitally corrected transposition of the great arteries (ccTGA) is a very rare and complex form of congenital heart disease.
2) Cardiac conduction disorders are well-established complications of ccTGA.
3) The presence or absence of associated cardiac disorders is an important parameter to determinate the risk of chronic heart failure.

## 1 Introduction

Congenitally corrected transposition of the great arteries (ccTGA) is a rare entity. As suggested by its name, It's characterized by atrioventricular and ventriculoarterial discordance. It's representing less than 0.05% of congenital heart disease lesions. Its incidence is estimated to be around 1/33.000 births [1]. The etiology of ccTGA remains unknown but an increased incidence of this disorder among families with history of previous cases suggests that genetic factors may be incriminated [2]. Cardiac conduction disorders are well-established complications of ccTGA [3]. It can be explained by the unusual position of the atrioventricular node and the circuit of the atrioventricular conduction bundle. Due to the improved survival of patients with congenital heart disease, it has become necessary for cardiologists to be able to diagnose this pathology, and even more important, to predict and manage its complications.

Our case report was written according to CARE guidelines [4].

## 2 Case presentation

A 19 year old patient presented to Ibn Sina University Hospital with episodes of lipothymia on exertion that appeared 3 weeks before his admission.

**Fig. 1:**
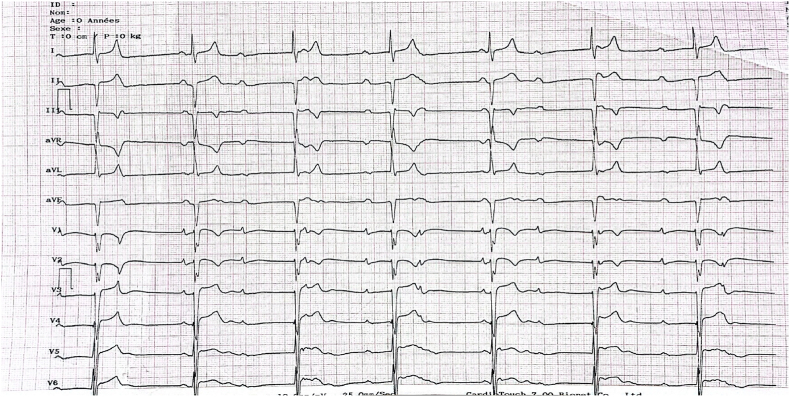
ECG showing complete atrioventricular block with a ventricular beat of 40/min.

**Fig. 2:**
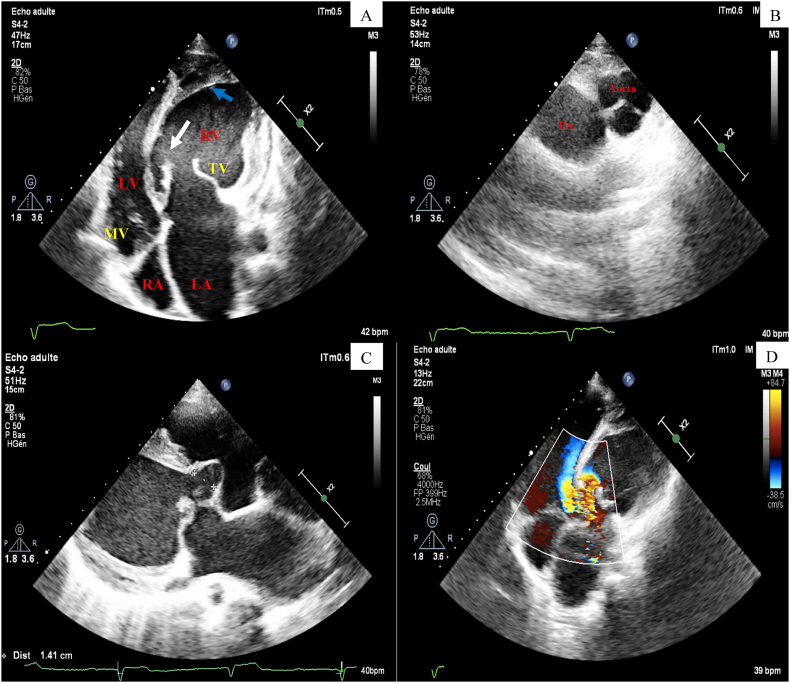
TEE findings: (A) Apical four chamber view showing the aspect of double discordance. Note the septal insertion of the left atrioventricular (AV) valve (white arrow) which is slightly apical compared to the right AV. The moderator band (blue arrow) can also be seen in the left sided ventricle. (B) Short axis view showing the anterior and left location of the aortic valve. (C) Modified parasternal long axis view showing a large perimembranous ventricular septal defect closed by spontaneously by a septal aneurysm. (D) Apical four chamber view showing tricuspid regurgitation that was quantified as moderate regurgitation and jet from the left sided ventricle to the right sided ventricle. (For interpretation of the references to colour in this figure legend, the reader is referred to the Web version of this article.)

No particular medical history was noted. The patient was conscious. His blood pressure was 140/80 mm Hg, his heart rate was 39 b/m and oxygen saturation value 98% on room air. Grade 3 systolic murmur was audible at the apex associated with a

a loud second heart sound. The electrocardiogram (ECG) showed complete atrioventricular block with a ventricular beat of 40/min (Fig. 1). Chest X-ray found a cardiomegaly with a double density sign consistent with left atrial enlargement. A transthoracic echocardiogram revealed inversion of the position of the 2 ventricles, according to the following schema: the left atrium connected, through the tricuspid valve, to a trabeculated left sided right ventricle and supplying systemic circulation through aorta. The right atrium connected, through the mitral valve, to a smooth-walled right sided left ventricle and supplying pulmonary circulation (Fig. 2-A). We noted marked enlargement of the left atrium and the left sided right ventricle with a moderate tricuspid regurgitation (Fig. 2-D). Systemic ventricular ejection fraction was normal. In addition to that, we observed a large perimembranous ventricular septal defect partially closed spontaneously by a septal aneurysm and responsible of a moderate right ventricular outflow obstruction (Fig. 2-C). Pulmonary artery systolic pressure (PASP) was estimated to be 50 mmHg assuming a right atrial pressure of 5 mmHg and pulmonary artery diastolic pressure was suggestive of low pulmonary resistance. Analysis of the right ventricle and tricuspid insufficiency by cardiac MRI was planned after discharge.

**Fig. 3:**
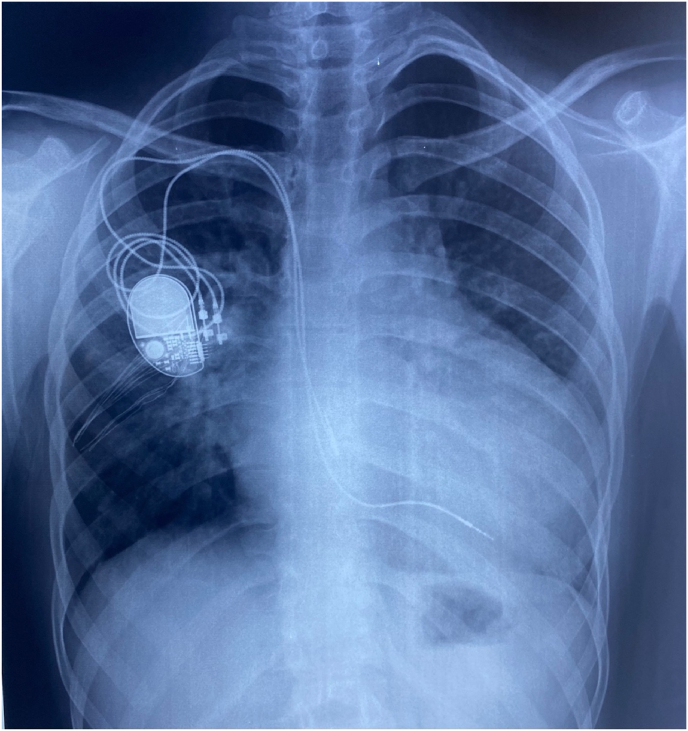
Chest x-ray showing dual-chamber pacemaker placed via the right subclavian vein, demonstrating correct position of the leads.

Cardiac resynchronization therapy (CRT) was indicated but due to financial constraints, a transvenous dual-chamber permanent pacemaker was implanted via right subclavian vein (Fig. 3). The initial outcome was favorable and the follow up was without particularities.

## 3 Discussion

Congenitally corrected transposition of the great arteries (ccTGA) is a rare congenital disorder. Embryologically, it can be explained by the abnormal left looping of the primitive heart. As result, the deoxygenated venous blood received from the superior and inferior vena cava passes into the right atrium and then to the discordant left ventricle through the mitral valve and finally into the lung through the discordant transposed pulmonary arteries. Once oxygenated, blood reaches the left atrium through the pulmonary veins and then into the discordant right ventricle via the tricuspid valve and finally returns to the systemic circulation through the discordant aorta.

The diagnosis of ccTGA can be made in adulthood incidentally, usually after a heart murmur, chest x-ray, ECG or echocardiography done for another reason [5]. Patients with ccTGA are exposed to two major risks: heart failure due to the progressive decline in systemic right ventricular function and cardiac conduction disorders, that may be seen at any age from fetal life to late adulthood. Indeed, Huhta et al. noted that the annual risk of developing AV block was 2% [6]. It reaches a prevalence of 30% in adulthood which is consistent with the case of our patient who presented a complete heart block at the age of 19 years old. This can be related to the abnormal position of the atrioventricular node and the course of the bundle of His.

It's important to emphasize that the presence or absence of associated cardiac disorders is an important parameter to determinate the risk of chronic heart failure. That was confirmed by Graham et al. who reported that by the age of 45 years, chronic heart failure was present in 67% of ccTGA patients with associated cardiac disorders [7]. In our case, we noted a perimembranous ventricular septal defect partially closed spontaneously by a septal aneurysm which implies a close monitoring of the right ventricular function of our patient.

Due to the unusual orientation of the cardiac chambers and the atrioventricular valves, the complexity of pacemaker implantation is increased. Furthermore, in a recent article published in The Journal of Thoracic and Cardiovascular Surgery, authors have reported that pacemaker implantation can be responsible of the deterioration of systemic ventricular function and worsening systemic atrioventricular valve regurgitation [8]. Accordingly, 2020 ESC Guidelines for the management of adult congenital heart disease suggest that biventricular pacing should be considered in case of complete AV block [9]. With regard to patients with severe systemic tricuspid regurgitation, they should be referred for tricuspid valve replacement (as repair is rarely feasible) before RV failure (ejection fraction less than 40%) [9].

## 4 Conclusion

The population of patients with adult congenital heart disease is constantly growing.

Due to the different clinical presentations and confusion with the appearance of “non-compaction” of the right systemic ventricle, the diagnosis of ccTGA remains challenging. Cardiologists must remain vigilant in order to establish an early diagnosis and ensure suitable management.

## Ethical approval

The ethical committee approval was not required give the article type (case report).However, the written consent to publish the clinical data of the patients was given and is available to check by the handling editor if needed.

## Sources of funding

None.

## Author contributions

Raid Faraj: Study concept, Data collection, Data analysis, Literature research, Writing the paper.

Abakar Bachar: Data collection, Data analysis.

Oussama Sidaty: Data collection, Data analysis.

Asmaa Bouamoud: Data collection, Data analysis.

Zineb Fassi Fehri: Data collection, Data analysis.

Fatima-Zahrae Chrifi: Data collection, Data analysis.

Fatima Chikhi: Supervision and data validation.

Ibtissam Fellat: Supervision and data validation.

Rachida Amri: Supervision and data validation.

Mohamed Cherti: Supervision and data validation.

## Trial registry number

This is not an original research project involving human participants in an interventional or an observational study but a case report. This registration is was not required.

## Guarantor

Raïd Faraj.

## Consent

Obtained.

## Provenance and peer review

Not commissioned, externally peer-reviewed.

## Declaration of competing interest

None.
